# Genomic characterization of multidrug‐resistant ESBL‐producing *Escherichia coli* ST58 causing fatal colibacillosis in critically endangered Brazilian merganser (*Mergus octosetaceus*)

**DOI:** 10.1111/tbed.13686

**Published:** 2020-07-02

**Authors:** Danny Fuentes‐Castillo, Pedro Enrique Navas‐Suárez, Maria Fernanda Gondim, Fernanda Esposito, Carlos Sacristán, Herrison Fontana, Bruna Fuga, Camila Piovani, Robert Kooij, Nilton Lincopan, José Luiz Catão‐Dias

**Affiliations:** ^1^ Department of Pathology School of Veterinary Medicine and Animal Sciences University of São Paulo São Paulo Brazil; ^2^ One Health Brazilian Resistance Project (OneBR) São Paulo Brazil; ^3^ Zooparque Itatiba Itatiba Brazil; ^4^ Department of Clinical Analysis Faculty of Pharmacy University of São Paulo São Paulo Brazil; ^5^ Department of Microbiology Instituto de Ciências Biomédicas University of São Paulo São Paulo Brazil

**Keywords:** bacterial infection, enterobacterales, ESBL, virulence, waterfowl, wildlife

## Abstract

Even though antimicrobial‐resistant bacteria have begun to be detected in wildlife, raising important issues related to their transmission and persistence of clinically important pathogens in the environment, little is known about the role of these bacteria on wildlife health, especially on endangered species. The Brazilian merganser (*Mergus octosetaceus*) is one of the most threatened waterfowl in the world, classified as Critically Endangered by the International Union for Conservation of Nature. In 2019, a fatal case of sepsis was diagnosed in an 8‐day‐old Brazilian merganser inhabiting a zoological park. At necropsy, major gross lesions were pulmonary and hepatic congestion. Using microbiologic and genomic methods, we identified a multidrug‐resistant (MDR) extended‐spectrum β‐lactamase (ESBL) CTX‐M‐8‐producing *Escherichia coli* (designed as PMPU strain) belonging to the international clone ST58, in coelomic cavity, oesophagus, lungs, small intestine and cloaca samples. PMPU strain harboured a broad resistome against antibiotics (cephalosporins, tetracyclines, aminoglycosides, sulphonamides, trimethoprim and quinolones), domestic/hospital disinfectants and heavy metals (arsenic, mercury, lead, copper and silver). Additionally, the virulence of *E. coli* PMPU strain was confirmed using a wax moth (*Galleria mellonella*) infection model, and it was supported by the presence of virulence genes encoding toxins, adherence factors, invasins and iron acquisition systems. Broad resistome and virulome of PMPU contributed to therapeutic failure and death of the animal. In brief, we report for the first time a fatal colibacillosis by MDR ESBL‐producing *E. coli* in critically endangered Brazilian merganser, highlighting that besides colonization, critical priority pathogens are threatening wildlife. *E. coli* ST58 clone has been previously reported in humans, food‐producing animals, wildlife and environment, supporting broad adaptation and persistence at human–animal–environment interface.

## 1 INTRODUCTION

Antimicrobial resistance (AMR) is one of the major Global Health challenges of the 21st century, and annually kills thousands of people in the world (Cassini et al., 2019; Hernando‐Amado, Coque, Baquero, & Martínez, 2019). One Health and Global Health approaches are necessaries to combat the emergence, evolution and spread of AMR (Hernando‐Amado et al., 2019). In this regard, wildlife has been suggested as reservoirs, disseminators or bio‐indicators of AMR in the environment (Borges et al., 2017; Dolejska & Literak, 2019; Sacristán et al., 2020); however, threatened wildlife species are being colonized by antibiotic‐resistant bacteria, but there are critical data gaps and research needs to understand the role and the real impact of AMR on wildlife (Fuentes‐Castillo et al., 2020; Larsson et al., 2018; Ramey & Ahlstrom, 2020).

The Brazilian merganser (*Mergus octosetaceus* Vieillot, 1817) is one of the most threatened avian species in the Americas and one of the most threatened waterfowl in the world, classified as Critically Endangered by the International Union for Conservation of Nature (BirdLife International, 2019; Lamas & Lins, 2009). It is estimated that its population does not exceed 250 mature individuals in nature but, thanks to conservation breeding programs, it has been possible to successfully reproduce the species ex‐situ (BirdLife International, 2019).

In this study, using microbiological and whole genome sequencing tools, we investigated a fatal sepsis caused by an antibiotic‐resistant bacterium in a critically endangered Brazilian merganser. In this regard, the resistome (antibiotics, heavy metals, and disinfectants), virulome and epidemiological characteristics of the pathogen were analysed.

## 2 MATERIALS AND METHODS

### 2.1 Brazilian merganser

As part of the Brazilian merganser Conservation Program, the Itatiba Zoological Park (Sao Paulo state, Brazil) carries out a successful breeding project. In October 2019, an 8‐day‐old Brazilian merganser hatched in the breeding program became ill presenting respiratory symptoms (dyspnoea, prostration, hyporexia and weight loss). The duck received prophylactic fluoroquinolone (i.e. Enrofloxacin, 15 mg/kg, IM, q. 12 hr), with unsuccessful results. The animal died presenting incoordination and opisthotonos, <24 hr after the first clinical signs.

### 2.2 Necropsy and sampling

Full necropsy examination was carried out at the Laboratory of Wildlife Comparative Pathology, Department of Pathology, School of Veterinary Medicine and Animal Science of the University of São Paulo, Brazil, according to Matushima. Representative samples of major organs/tissues, including oesophagus, proventriculus, small and large intestines, pancreas, spleen, liver, lungs, trachea, heart, aorta and kidney, were collected and fixed in 10% neutral buffered formalin. Central nervous system was not sampled to preserve the cranium for museum collection. Tissue samples were processed routinely and embedded in paraffin wax. Sections (5 μm) were stained with haematoxylin and eosin. Additionally, selected samples from coelomic cavity, oral cavity, oesophagus, lungs, small intestine and cloaca were aseptically sampled using sterilized swabs and deposited in Amies transport medium with charcoal for posterior microbiological analysis.

### 2.3 Isolation, bacterial identification and antimicrobial susceptibility testing

Cloacal, coelomic and oral cavity and tissue swab samples were streaked onto blood and MacConkey agar plates and incubated overnight at 35 ± 2°C. Bacterial isolates were identified by the MALDI‐TOF MS system (Bruker Daltonik), and clonal relationships among *Escherichia coli* isolates were determined by enterobacterial repetitive intergenic consensus (ERIC)‐PCR (Da Silveira et al., 2002).

Antimicrobial susceptibility testing was performed by the disc diffusion method using human and veterinary antimicrobials (CLSI, 2018, 2020), including amoxicillin/clavulanate, ceftriaxone, cefotaxime, ceftiofur, ceftazidime, cefepime, cefoxitin, aztreonam, imipenem, meropenem, ertapenem, nalidixic acid, enrofloxacin, gentamicin, amikacin, trimethoprim‐sulfamethoxazole and tetracycline. *E. coli* ATCC 25922 was used as control strain. Extended‐spectrum β‐lactamase (ESBL) production was screened by the double‐disc synergy test (DDST; Jarlier, Nicolas, Fournier, & Philippon, 1988).

### 2.4 Whole genome sequence (WGS) analysis

For selected ESBL‐producing *E. coli* strain, genomic DNA was extracted using a PureLinkTM Quick Gel Extraction Kit (Life Technologies), and a genomic paired‐end library (75 × 2 bp) was prepared using a Nextera XT DNA Library Preparation Kit (Illumina Inc.) according to the manufacturer's instructions. The whole genome was sequenced on the NextSeq platform (Illumina). De novo genome assembly and contig annotation was carried out using CLC Genomics Workbench 12.0.3. Multilocus sequence type (MLST), plasmid replicons, resistome and serotype were identified using MLST v2.0 (Larsen et al., 2012), PlasmidFinder v2.1 (Carattoli et al., 2014), ResFinder v3.2 (Zankari et al., 2012) and SerotypeFinder v2.0 (Jenkins, 2015) tools, respectively, from Center for Genomic Epidemiology (http://www.genomicepidemiology.org/). Clinically, important virulence factors were detected and compared by ABRicate v0.9.8 (https://github.com/tseemann/abricate) using data from the *Escherichia coli* Virulence Factors (https://github.com/phac‐nml/ecoli_vf) and the Virulence Factor Database (VFDB; http://www.mgc.ac.cn/VFs/). Heavy metal (HM) and biocides genes were detected using the BacMet2 experimentally confirmed database (http://bacmet.biomedicine.gu.se). For whole genome of selected ESBL‐producing *E. coli* identified in this study, a minimum spanning tree was constructed in Enterobase using the MSTree V2 algorithm and the wgMLST scheme (https://enterobase.warwick.ac.uk/species/index/ecoli). This scheme consists of 25,002 pan‐genome genes present in *E. coli* genomes, which represented most of the diversity in Enterobase at the time (March 2020; https://bitbucket.org/enterobase/enterobase‐web/wiki/Escherichia%20Statistics). All images were generated with iTOL v.5.5 (https://itol.embl.de).

### 2.5 In vivo virulence assays in the greater wax moth (*Galleria mellonella*) infection model

In vivo virulence behaviour of ESBL‐producing *E. coli* was evaluated using the *G. mellonella* infection model (Tsai, Loh, & Proft, 2016). The non‐virulent *E. coli* ATCC 25922 and the hypervirulent meningitis/sepsis‐associated K1 *E. coli* strain (MNEC RS218; Achtman et al., 1983; Santos, Zidko, Pignatari, & Silva, 2013) were used as non‐virulent and hypervirulent controls. In brief, *G. mellonella* larvae, of nearly 250 to 350 mg, were inoculated with 10^5^ CFU of each strain. Survival of two *G. mellonella* groups (each group composed by 20 larvae) inoculated with each strain were evaluated for 96 hr. Data were analysed by the log rank test, with *p* < .05 indicating statistical significance (Prism GraphPad Software).

## 3 RESULTS AND DISCUSSION

### 3.1 Pathological findings

**FIGURE 1:**
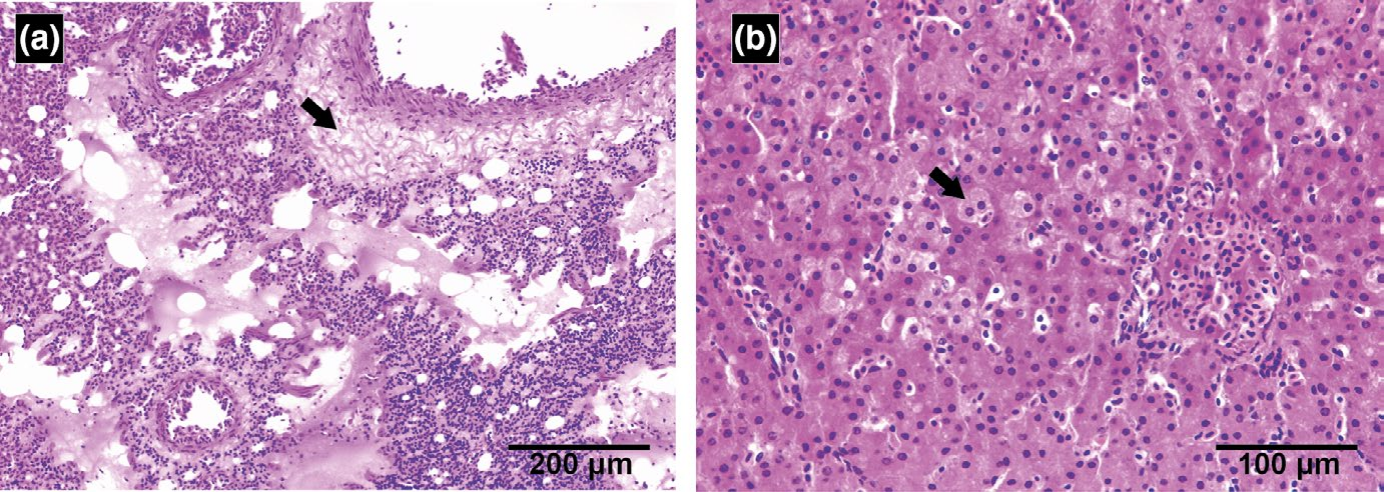
Microscopic findings in an 8‐day‐old Brazilian merganser (*Mergus octosetaceus*) with colibacillosis. In (a) Lungs, note congestion of alveolar capillaries and perivascular oedema (black arrow). In (b) Liver, note hepatocellular swelling and intracytoplasmic vacuolation (black arrow). Haematoxylin and eosin staining

The main gross finding was dark reddish coloration in the lungs, draining a marked amount of serosanguineous fluid. Microscopically, haemodynamic disturbances were observed in the lungs, highlighting a marked congestion of blood vessels and alveolar capillaries, and mild acute alveolar haemorrhage (Figure 1a). In liver, moderate congestion in zone I and II was detected (Figure 1b). Finally, in kidney, corticomedullar congestion was also observed. Histopathological alterations were not perceived in the remaining organs/tissues analysed.

### 3.2 Bacterial isolation, identification and antimicrobial resistance profile

*Escherichia coli* was isolated from coelomic cavity, oesophagus, lungs, small intestine and cloaca. Clonal relatedness analysis (ERIC‐PCR) and antimicrobial resistance profile confirmed a systemic infection by an identical *E. coli* clone, compatible with avian colibacillosis (Díaz‐Sánchez et al., 2013; Kabir, 2010; Maciel et al., 2017; Sarowska et al., 2019). All *E. coli* strains were ESBL producers and displayed a resistant profile to human and veterinary broad‐spectrum cephalosporins, tetracyclines, aminoglycosides, sulphonamides, trimethoprim and quinolones, remaining susceptible to carbapenems, cephamycin and monobactams. An *E. coli* strain isolated from the lung tissue was randomly selected to WGS analysis and designed as PMPU strain.

### 3.3 *E. coli* PMPU strain carried a wide resistome to antibiotics, heavy metals, and disinfectants

**FIGURE 2:**
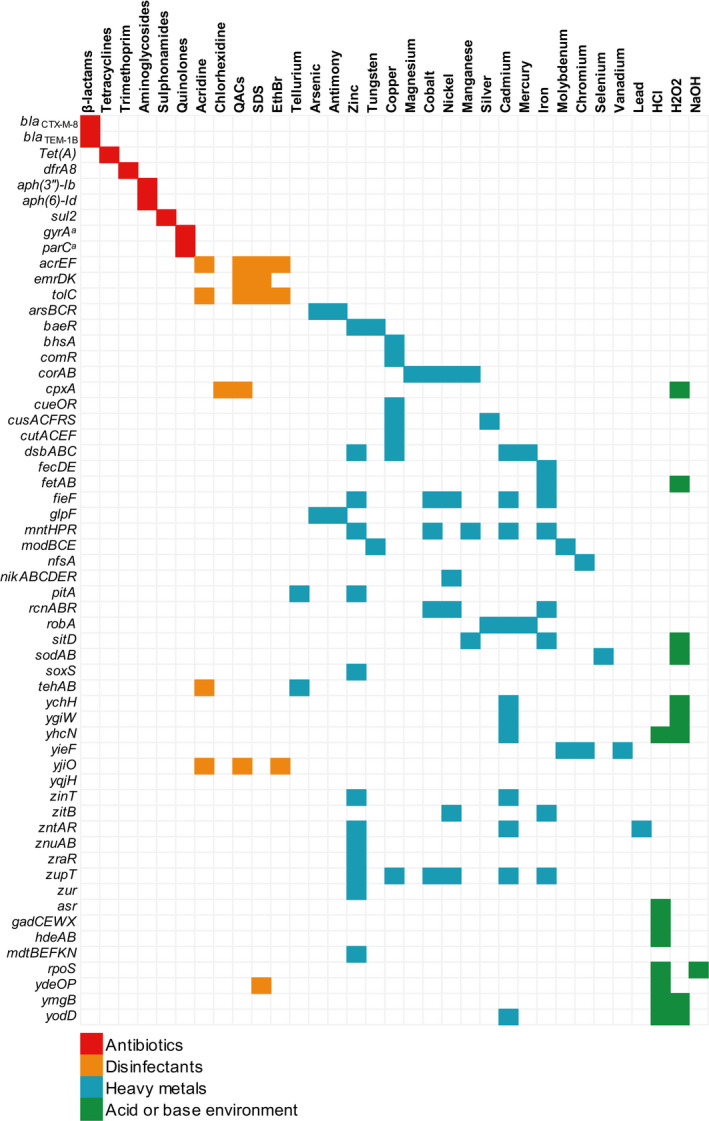
Resistome of multidrug‐resistant CTX‐M‐8‐producing *Escherichia coli* PMPU strain. Infographic shows the names of detected genes in *E. coli* PMPU whole genome (rows), which encode resistance to antibiotics, disinfectants, heavy metals, and acid or basic environment (columns). ^a^Mutations in quinolone resistance‐determining region (QRDR). EthBR, ethidium bromide; H2O2, hydrogen peroxide; HCl, hydrochloric acid; NaOH, sodium hydroxide; QACs, quaternary ammonium compounds; SDS, sodium dodecyl sulfate

PMPU strain belonged to sequence type ST58 and serotype O102:H30. This strain harboured a resistome against antibiotics, heavy metals and disinfectants. WGS analysis identified the presence of genes encoding resistance to cephalosporins (*bla*_CTX‐M‐8_ and *bla*_TEM‐1B_), tetracyclines [*tet(A)*], aminoglycosides [*aph(3″)Ib* and *aph(6)‐Id*], sulphonamides (*sul2*) and trimethoprim (*dfrA8*). In addition, the PMPU strain displayed mutations in *gyrA* (Ser‐83‐Leu and Asp‐87‐Asn) and *parC* (Ser‐80‐Iso) genes, which confer resistance to fluoroquinolones, causing therapeutic failure when enrofloxacin was used as prophylactic treatment in the animal. Moreover, genes conferring resistance to heavy metals (i.e. lead, arsenic, copper, silver, antimony, zinc, tellurium, tungsten, magnesium, cobalt, nickel, manganese, cadmium, mercury, iron, molybdenum, chromium, selenium and vanadium) and biocides commonly used as disinfectants in domiciliary and hospital settings (i.e. quaternary ammonium compounds [QACs], acridines, chlorhexidine, sodium dodecyl sulphate, ethidium bromide, hydrochloric acid, hydrogen peroxide and sodium hydroxide) were found (Figure 2). Regarding to plasmidome in PMPU strain, IncI1 and IncQ1 plasmid replicons were detected.

*Escherichia coli* ST58 is a globally disseminated clone previously reported in humans, food‐production animals, wildlife and the environment, supporting a broad adaptation, persistence and a worldwide dissemination of this clone (Borges, Tarlton, & Riley, 2019; De Carvalho et al., 2020; EnteroBase, 2020; McKinnon, Roy Chowdhury, & Djordjevic, 2018; Zurfluh et al., 2019). In Brazil, *E. coli* ST58 has been isolated from humans, poultry, peri‐urban wild animals and polluted mangrove ecosystem (Borges et al., 2019; De Carvalho et al., 2020; Sacramento et al., 2018). On the other hand, MDR or ESBL‐producing *E. coli* serotype O102:H30 has been recurrently identified in hospitalized human patients, mainly with urinary tract infection (Cergole‐Novella, Guth, Castanheira, Carmo, & Pignatari, 2010; Cergole‐Novella, Pignatari, & Guth, 2015; Gonçalves et al., 2009).

**FIGURE 3:**
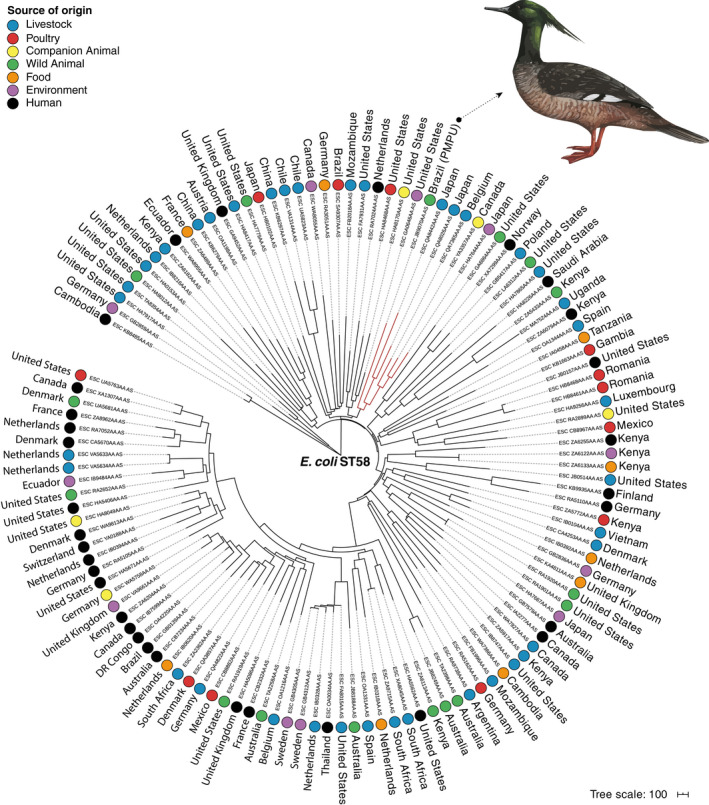
Phylogeny of CTX‐M‐8‐producing *Escherichia coli* isolate from a Brazilian merganser (*Mergus octosetaceus*), in relation to an international *E. coli* collection. The image shows a minimum spanning tree based on wgMLST of 123 worldwide distributed *E. coli* strains belonging to ST58, constructed by the MSTree V2 tool from EnteroBase. The figure was generated with iTOL v.5.5 (https://itol.embl.de). Interactive versions of the tree can be found at https://itol.embl.de/tree/20014463144294501588789515. Coloured circles represent sources of origin. Each isolate is indicated by the country of origin

We further investigated the genomic relatedness among *E. coli* PMPU isolate identified in this study and 123 assembled genomes of *E. coli* belonging to ST58 from different sources of origin and countries, available in EnteroBase database (https://enterobase.warwick.ac.uk/). In the minimum spanning tree of the whole genome analysis based on the wgMLST scheme from EnteroBase, *E. coli* PMPU isolate showed high genetic relatedness compared to livestock isolates from Japan (ESC_QA8442AA_AS and ESC_QA8026AA_AS) and Belgium (ESC_QA7365AA_AS), an animal companion isolate from Canada (dog; ESC_YA3357AA_AS) and an environment isolate from Japan (ESC_HA7644AA_AS; Figure 3). These phylogenetically related isolates were collected between 2013 and 2018, supporting rapid adaptation and dissemination of this *E*. *coli* clone. The importation and spread of ESBL‐producing Enterobacterales between geographically distant countries has been attributed to international travel people (Arcilla et al., 2017; Armand‐Lefèvre, Andremont, & Ruppé, 2018; Frost, Van Boeckel, Pires, Craig, & Laxminarayan, 2019), international imports of live animals or raw meat (Mo et al., 2014; Nahar et al., 2018; Schaumburg et al., 2014) and migratory wild birds (Báez et al., 2015). This could explain the presence of phylogenetically related *E. coli* ST58 clones that circulate between countries on different continents.

### 3.4 Virulome of ESBL‐positive *E. coli* ST58 colonizing Brazilian merganser is associated with a virulent behaviour

Virulome analysis of ESBL‐producing *E. coli* PMPU strain highlighted virulence factors, including adherence factors (*fim, eaeH*, *lpfAO113*, *csgBCDEFG*), invasins (*iss*, *ibeBC*), cytolytic pore‐forming toxin (*hlyE*), iron acquisition systems (*entBCEFS*, *fepABCD*) and chemotaxis (*cheABRMWYZ*, *motAB*), among other virulence factors commonly found in commensal and pathogenic *E. coli* strains (Table 1). The virulent potential of PMPU strain was confirmed in the *G. mellonella* infection model, where strains inoculated at 1 × 10^5^ CFU killed 100% of wax moth larvae within 50h, showing a more virulent behaviour than *E. coli* ATCC 25922, but no more than hypervirulent meningitis‐causing *E. coli* MNEC RS218 (Figure S1). *G. mellonella* has been successfully utilized as an in vivo model to assess the pathogenic potential of clinically important bacterial pathogen. Therefore, responses to bacterial infections observed in this model could closely mimics responses displayed by mammalian models (Jander, Rahme, & Ausubel, 2000; Kavanagh & Reeves, 2004; Lange et al., 2018). In this study, virulent performance of *E. coli* PMPU strain was correlated with virulence factors commonly identified in pathogenic *E. coli* lineages from humans and poultry, highlighting adherence factors (*fimBCEFGHI, eaeH*, *lpfAO113*, *csgBCDEFG*; Dale & Woodford, 2015; Osek, Weiner, & Hartland, 2003; Sarowska et al., 2019; Torres, 2016), invasins (*iss*, *ibeBC*; Sarowska et al., 2019), toxin (*hlyE*; Wyborn et al., 2004), iron acquisition systems (*entBCEFS*, *fepABCD*; Robinson, Heffernan, & Henderson, 2018; Torres, 2016) and chemotaxis factors (*cheABRMWYZ*, *motAB*; Pettersen, Mosevoll, Lindemann, & Wiker, 2016). In this regard, adherence factors and invasins found in the *E. coli* PMPU strain may have contributed to the colonization in different tissues of the bird; the cytolytic pore‐forming toxin *hlyE* could be related to haemodynamic disturbances and tissue damage found in the histopathology (Lai et al., 2000; Lithgow, Haider, Roberts, & Green, 2007; Oscarsson et al., 1999). On the other hand, the immature immune system in an 8‐day‐old Brazilian merganser, the artificial incubation conditions (Ruiz‐Castellano, Tomás, Ruiz‐Rodríguez, Martín‐Gálvez, & Soler, 2016), as well as use of disinfectants, may contributed to the selection of a virulent *E. coli* resistant to a wide range of antibiotics and disinfectants, establishing a disseminated infection with a fatal end. In order to avoid new infections due to *E. coli* widely resistant to antimicrobials and disinfectants, a cleaning of the environments was carried out using peracetic acid concentrated at 0.2%. After this occurrence, no new cases of fatal infection caused by this pathogen were registered in animals at this zoological park.

**TABLE 1:** Virulome of MDR CTX‐M‐8‐producing *Escherichia coli* PMPU strain isolated from haemorrhagic pulmonary tissue of an 8‐day‐old Brazilian Merganser

Characteristics	Virulence genes
Adherence	
Fimbriae	*fimBCEFGHI*, *cfaABCD*, *lpfAO113*, *matF*, *stgBCD*, *ycbFRSTUV*
Flagella	*flgABCDEFGHIJKLN*, *flhABCDE*, *fliADEFGHIJKLMNOPQRSTYZ*, *flk*
Pilus	*hofCB*
Adherence haemorrhagic coli pilus	*ppdABCD*, *hofQ*, *ygdB*, *yggR, b2854*, *b2972*
Adhesins	*eaeH*, *ecpRABCD*, *ehaABG*
Curli fibres	*csgBCDEFG*
Protectins and invasins	
Colicin	*cib*
Increased serum survival	*iss*
Invasin	*ibeBC*
Iron acquisition systems	
Enterobactin	*entBCEFS*, *fes*
Ferrienterobactin	*fepABCD*
Toxins	
Haemolysin E	*hlyE*
Secretion systems components	
Type II secretion system	*gspCDEFGHIJKLM*, *yghG*
Type III secretion system	*espL3−4, espR1*, *espX1−5*, *eprHIJK*
Others	
Glutamate decarboxylase	*gadX*
Lysine decarboxylase	*cadA*
Chemotaxis	*cheABRMWYZ*, *motAB*
Surface presentation of antigens	*epaOPQRS*

Virulent pathogens resistant to an increasing number of antimicrobials cause thousands of deaths in the human population each year (Cassini et al., 2019; Centers for Disease Control, 2019; Gu et al., 2018). In this concern, wildlife plays an important role in the epidemiology of antibiotic‐resistant pathogens in the environment (Alcalá et al., 2016; Sevilla et al., 2020; Vittecoq et al., 2016). However, little is known about the impact of these MDR pathogens on wildlife, especially on threatened wildlife species (Gonçalves et al., 2012; Larsson et al., 2018; Ramey & Ahlstrom, 2020). In this study, we isolated a MDR ESBL‐producing *E. coli* with virulent behaviour, belonging to international clone ST58 and serotype O102:H30, causing fatal infection in a critically endangered Brazilian merganser. Of note, a MDR colistin‐resistant *E. coli* ST58 was recently isolated from a polluted mangrove ecosystem in Brazil (Sacramento et al., 2018); therefore, a similar biological threat may potentially be disseminated among humans and wildlife via environmental pathways.

Although virulent characteristics of *E. coli* PMPU strain, and dissemination findings through different organs are compatible with a fatal avian colibacillosis, absence of investigations on non‐bacterial pathogens were limitations for this study.

A better integration of environmental and wildlife issues is necessary to a successful One Health approach for global AMR crisis (White & Hughes, 2019). In this context, to understand epidemiologically the evolution and adaptation of AMR, wildlife veterinarians must increasingly report the challenges that arise when treating antimicrobial‐resistant pathogenic bacteria in wildlife species. Herein, we report a fatal colibacillosis by a MDR ESBL‐producing *E. coli* in critically endangered Brazilian merganser, highlighting that besides colonization, antimicrobial‐resistant pathogens are threatening wildlife health.

## CONFLICT OF INTERESTS

No potential conflict of interest was reported by the authors.

## ETHICAL APPROVAL

The authors confirm that the ethical policies of the journal, as noted on the journal's author guidelines page, have been adhered to. No ethical approval was required for this specific study.

## Supporting information

Figure S1

## Data Availability

The whole genome nucleotide sequence of the *E. coli* PMPU isolate is available in the GenBank database under accession number PRJNA608189. In addition, genomic data of E. coli PMPU strain is available in the OneBR project, under the number ID ONE107 (http://onehealthbr.com/).
