# Supplementary material for: Genomic characterization of multidrug‐resistant ESBL‐producing Escherichia coli ST58 causing fatal colibacillosis in critically endangered Brazilian merganser (Mergus octosetaceus)
Source: Transbound Emerg Dis. 2020 Jul 2;68(2):258–66. doi: 10.1111/tbed.13686 (PMC8246901; doi:10.1111/tbed.13686)
Supplement: Supplementary file 1 — Figure S1 [file TBED-68-258-s001.docx]

**Supplementary information**

**Genomic characterization of multidrug-resistant ESBL-producing *Escherichia coli* ST58 causing fatal colibacillosis in critically endangered Brazilian merganser (*Mergus octosetaceus*)**

Danny Fuentes-Castillo, Pedro Enrique Navas-Suárez, Maria Fernanda Gondim, Fernanda Esposito, Carlos Sacristán, Herrison Fontana, Bruna Fuga, Camila Piovani, Robert Kooij, Nilton Lincopan, José Luis Catão-Dias

**Figure S1.**

**
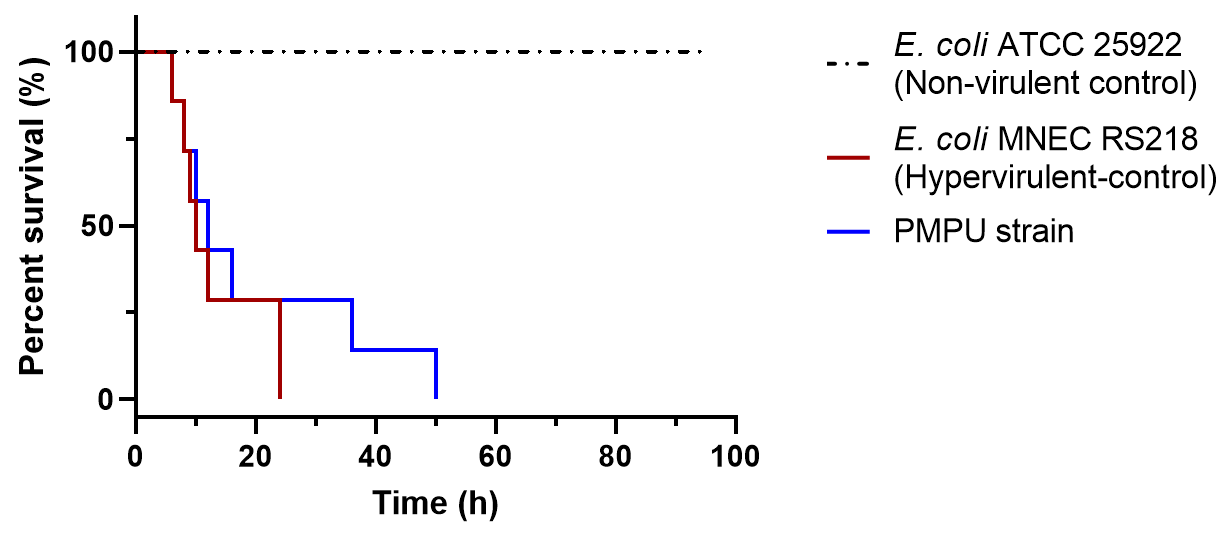
**

Survival curves of greater wax moth (*Galleria mellonella*) larvae after infection with 10^5^ CFU/larva of ESBL-producing *Escherichia coli* isolated from a Brazilian merganser (*Mergus octosetaceus*), non-virulent *E. coli* ATCC 25922 and highly virulent *E. coli* MNEC RS218 strains. ESBL-producing *E. coli* PMPU strain killed 100% of wax moth larvae within 50 h, showing a more virulent behavior than *E. coli* ATCC 25922, but no more than meningitis-causing *E. coli* MNEC RS218.
